# Microbe-Mediated Thermotolerance in Plants and Pertinent Mechanisms- A Meta-Analysis and Review

**DOI:** 10.3389/fmicb.2022.833566

**Published:** 2022-03-07

**Authors:** Khondoker M. G. Dastogeer, Mst. I. Zahan, Mohammad S. Rhaman, Mohammad S. A. Sarker, Anindita Chakraborty

**Affiliations:** ^1^Department of Plant Pathology, Bangladesh Agricultural University, Mymensingh, Bangladesh; ^2^Scientific Officer (Breeding Division), Bangladesh Sugarcrop Research Institute, Pabna, Bangladesh; ^3^Department of Seed Science and Technology, Bangladesh Agricultural University, Mymensingh, Bangladesh; ^4^Basic and Applied Research on Jute Project, Bangladesh Jute Research Institute (BJRI), Dhaka, Bangladesh; ^5^Department of Genetic Engineering and Biotechnology, Shahjalal University of Science and Technology, Sylhet, Bangladesh

**Keywords:** antioxidant, heat stress, meta-analysis, plant-microbe interaction, plant physiology, symbiosis

## Abstract

Microbial symbionts can mediate plant stress responses by enhancing thermal tolerance, but less attention has been paid to measuring these effects across plant-microbe studies. We performed a meta-analysis of published studies as well as discussed with relevant literature to determine how the symbionts influence plant responses under non-stressed versus thermal-stressed conditions. As compared to non-inoculated plants, inoculated plants had significantly higher biomass and photosynthesis under heat stress conditions. A significantly decreased accumulation of malondialdehyde (MDA) and hydrogen peroxide (H_2_O_2_) indicated a lower oxidation level in the colonized plants, which was also correlated with the higher activity of catalase, peroxidase, glutathione reductase enzymes due to microbial colonization under heat stress. However, the activity of superoxide dismutase, ascorbate oxidase, ascorbate peroxidase, and proline were variable. Our meta-analysis revealed that microbial colonization influenced plant growth and physiology, but their effects were more noticeable when their host plants were exposed to high-temperature stress than when they grew under ambient temperature conditions. We discussed the mechanisms of microbial conferred plant thermotolerance, including at the molecular level based on the available literature. Further, we highlighted and proposed future directions toward exploring the effects of symbionts on the heat tolerances of plants for their implications in sustainable agricultural production.

## Introduction

Due to climate change, the constant rise of ambient temperature is one of the major global issues and has devastating impacts on crop growth and productivity, consequently, in food security. Results from various climate model simulations predict that the global average temperature could be between 1.1 to 5.4°C warmer in 2100 than it is today (IPCC, 2019). Heat stress causes a manifold adverse impact on the growth, development, physiological processes of plants (Wahid et al., 2007; Hasanuzzaman et al., 2013; Hassan et al., 2021; Jagadish et al., 2021). The plant employs varying levels of adaptation, avoidance, acclimation, and tolerance mechanisms to cope with heat stress *via* physical, physiological, biochemical, and molecular strategies, including the use of ion transporters, proteins, osmolytes, antioxidants, and other factors involved in signaling and transcriptional regulation (Hasanuzzaman et al., 2013; Hassan et al., 2021; Jagadish et al., 2021; Zhao et al., 2021). Plant responses to abiotic stress have been studied widely for the last few years, and the role of microbes on plant stress responses has also been given attention in recent years. In particular, plant species commonly associated with microbial symbionts, known as plant microbiome, may influence responses of the host plant to environmental stimuli, including heat stress.

Microorganisms are ubiquitous, and all plants are colonized by various types of microbes which play important roles in plant ecology and physiology (Rodriguez et al., 2009; Turner et al., 2013; Compant et al., 2019; Dastogeer et al., 2020a). For example, AMF (arbuscular mycorrhizal fungi) present in the roots of 80% of terrestrial plants (Brachmann and Parniske, 2006) and microbial endophytes (both fungal and bacterial) are highly diverse and live asymptomatically in every plant studied so far. It is increasingly recognized that these microbes, as well as others including, e.g., epiphytes, viruses, ectomycorrhiza, N-fixers, etc., exert multiple effects on the plants particularly under adverse environmental conditions (Porras-Alfaro and Bayman, 2011; Hill et al., 2019; Dastogeer et al., 2020b; Kumar et al., 2020). These beneficial effects, which are strongly dependent on environmental conditions (Rodriguez et al., 2009; Hoeksema et al., 2010; Dastogeer, 2018), include priming against pests or herbivores, the acquisition of growth-limiting nutrients from the soil, tolerance to drought, salinity and thermal stress, etc. (Rodriguez et al., 2009; Goh et al., 2013; Hardoim et al., 2015; Azad and Kaminskyj, 2016; González-Teuber, 2016; Molina-Montenegro et al., 2016; Dastogeer and Wylie, 2017; Rho et al., 2018; Hill et al., 2019). Microbes produce a wide range of compounds to impact the responses of plants at the molecular level, triggering the biosynthesis of pigments, secondary metabolites, hormones, antioxidants, and alkaloids (Meena et al., 2017; Dastogeer et al., 2018; Basit et al., 2021; Naamala and Smith, 2021). Just as for other stresses, it is becoming increasingly evident that the heat stress responses of plants may be influenced by their interactions with microbes (Eerens et al., 1998; Ali et al., 2011; Khan A. L. et al., 2015; Ismail et al., 2020a; Li X. et al., 2021).

Despite the ubiquity and high diversity of microbial symbionts in plant tissues and the widespread effects and economic relevance of heat as a stress factor for plants, when compared with other abiotic factors such as drought, salinity, or nutrient limitation, the involvement of symbionts in plant host responses to heat stress have been received less attention (Meena et al., 2017; Kumar and Verma, 2018). The handful of studies available indicate that the magnitudes of the effect of microbes on plant heat stress tolerance and associated mechanisms such as accumulation of osmolytes, antioxidants, phytohormones, etc., vary among various studies (Meena et al., 2017; Kumar and Verma, 2018). We assume these differences could be associated with multiple factors such as level of stress, types of host and symbiont partners, environmental conditions, and their complex interactions. In order to gain insight into and harness the benefit of symbiont in agricultural management practice, it is imperative to elucidate the detailed mechanism of microbe-plant interaction under heat stress conditions (Harman and Uphoff, 2019). It is indeed tenuous and often erroneous to deduce the findings in general context from the individual investigation (Dastogeer, 2018). Therefore, to ascertain the central tendency and pinpoint the patterns of microbial influence on plants under stress and compare with them under control, it is paramount to integrate results across studies in order to determine if the factors can be known (Borenstein et al., 2009; Dastogeer, 2018). Here, we performed a meta-analysis to measure the overall strength and direction of effect (beneficial, harmful or neutral) of symbiosis on important plant characteristics associated with stress tolerance mechanisms. To the best of our knowledge, this aspect has not previously been discussed in a review of the published article, and thus present article fills a substantial gap in our understanding of interactions between plants and their symbiotic microbial partners.

A meta-analysis is a mathematical and statistical approach that pools the results of various investigations to estimate a mean effect size for treatment across a range of studies (Rosenberg et al., 2000). We can evaluate the findings of a study with respect to all other comparable studies to assess if the effect of a treatment is consistent across studies or if it differs substantially among studies and which factor might be accounted for the differences (Borenstein et al., 2009). The categorical variables or “moderators” are often included in meta-analyses to pinpoint which and how various features modulate the treatment effect of interest. This form of analysis has been, for example, used to determine the impact of microbial symbionts on plant response to salinity stress (Chandrasekaran et al., 2014, 2016; Rho et al., 2018), drought stress (Dastogeer, 2018; Rho et al., 2018), and cold stress (Acuna-Rodriguez et al., 2020). In the current study, we accumulated data from all studies to date and measured the effects of endophytes on 24 plant response parameters that encompass plant growth, photosynthesis, metabolites, and enzymatic activities that are subjected to change under thermal stress conditions. Our purpose was to answer the questions, broadly; what is the overall impact of microbial colonization on various plant growth and photosynthetic parameters of plants exposed to heat stress? Does the plant-symbiont relationship differ under heat-stressed conditions compared to unstressed conditions? What is the role of symbiosis in osmotic balance and antioxidant enzymes regulation in plants exposed to thermal stress? We supplemented our meta-analysis with a review of relevant literature for a deeper insight into this subject.

## Literature Search and Study Selection for Meta-Analysis

The meta-data were collected by following the general guidelines of Field and Gillett (2010). We performed a literature search in Web of Science (Clarivate Analysis) and PubMed database through September 2020. Our search terms were: endophyte or AMF or mycorrhiza or bacteria or fungi AND either “heat stress*” or “hot stress*” or “high temperature.” The Boolean truncation (“*”) was used so as to include the variations of the word such as fungi, fungus, and fungal. The search produced 4,562 unduplicated papers, and 90 peer-reviewed articles were considered likely to contain relevant information by reading the title and abstract. To finally select the papers for data extraction, we had preset criteria such as so that these studies can be included in our meta-analysis and extreme heterogeneity minimized (Cooper, 2015; Ahn and Kang, 2018; Cooper et al., 2019; Seidler et al., 2019):

(i) The article must describe the findings of original research, and as such, review papers, opinion articles, editorials, book chapters, and systematic reviews were discarded for inclusion into our meta-analysis.
(ii) The investigation had to use at least one microbial strain regardless of inoculation protocol or rate,
(iii) The inoculum was used singly, and we did not consider any mixed-inoculation for this meta-analysis,
(iv) Both microbe-inoculated and non-inoculated plants were grown under normal stressed and non-stressed conditions, and data provided,
(v) Any of the growth and other parameters, e.g., biomass, enzymes, hormones, etc., were measured
(vi) The papers must have provided sample size (i.e., replication), means, standard deviations/errors, and other relevant statistical data that could be converted to measure an effect size.

Based on the above criteria, most articles were excluded for meta-analysis, and only 39 peer-reviewed original research articles were retained for the final analysis (Supplementary Table 1). We accepted studies to differ in the levels of fertilizer applied, growth conditions (greenhouse, growth chamber, or field), duration of stress application, the magnitude of stress, and growth media into our meta-analysis. Papers spanned 23 years (1998–2021) and were in English. We apologize for not including the potential paper that contains data written in other languages or those that we might have unintentionally missed during our search or due to stipulated selection criteria.

## Data Extraction and Meta-Analysis

When a publication provided results of more than one study system/group, those systems were taken as independent data points (Hedges et al., 1999). From these articles, we extracted information on host identity (plant species, genus, family), symbionts’ identity (genus, species), plant biomass (shoot and root), length (shoot and root), relative water content, photosynthetic parameters, enzymes parameters, and other relevant data from the studies (Supplementary Table 2). The means, sample sizes (replications), standard deviation were recorded from each study. Standard deviation was calculated from standard errors (SE) using the equation: SE = SD (n^–1/2^). Frequently the results were presented in a graph, and we used WebPlotDigitizer (Rohatgi, 2021) to digitize the values. If any paper reported multiple treatments or host/AMF combinations, these were included as an independent data unit in the analysis. However, it might increase the dependence of a particular study when considered them as an independent report (Gurevitch and Hedges, 1999). Nonetheless, this practice is assumed to increase the statistical power of meta-analysis (Lajeunesse and Forbes, 2003) and several biological meta-analyses papers also used the same (Holmgren et al., 2012; Veresoglou et al., 2012; Mayerhofer et al., 2013; McGrath and Lobell, 2013; Dastogeer, 2018).

Meta-analyses were conducted using the “metacont” function of the package “meta” (Balduzzi et al., 2019) implemented in R version 4.0.4 (R Core Team, 2021). Although the DerSimonian and Laird (DL) method is commonly used by default to estimate the between-study variance, it was suggested that for continuous data, the restricted maximum likelihood estimator (REML) are better alternatives to estimate the between-study variance (DerSimonian and Laird, 1986; Veroniki et al., 2016; Cooper et al., 2019). We used the Restricted maximum-likelihood estimator (REML) to make statistical inferences considering the effects as random. Standardized mean difference (SMD), which is defined as the ratio of the difference in mean performance between treatments to the pooled standard deviation, was calculated. This effect size measurement is done in meta-analyses that involve studies reporting continuous outcomes, similar to our study (Faraone, 2008). Generally, the SMD values of 0.3, 0.5, and 0.8 are considered as a small, medium, and large effect sizes, respectively (Cohen, 1988). Quantification of heterogeneity as well as testing the significance were estimated with Higgin’s I^2^ and Cochran’s Q statistics. “I^2^” is defined as the ratio of true heterogeneity to total heterogeneity across the effect sizes, whereas “Q” is the weighted deviations of the summary effect size that is due to true heterogeneity rather than sampling error (Higgins and Thompson, 2002; Higgins et al., 2003; Huedo-Medina et al., 2006). The I^2^ values < 25%, 25–75%, and > 75% are regarded as representing low, moderate, and high heterogeneity (Higgins et al., 2003) by convention. The coefficient of interval estimate of effect sizes was set to 95%, and the effect size (SMD) was considered significant when 95% CIs (confidence interval) did not cross zero. SMD of zero suggests that the two treatments (microbial inoculated or non-inoculated) have equivalent effects; SMDs greater than zero indicate the degree to which the microbial inoculated outperforms the non-inoculated samples and *vice-versa*.

Publication bias for each dataset of different parameters was tested by inspecting funnel plots and Egger regression test for funnel plot asymmetry (Begg and Mazumdar, 1994; Egger et al., 1997; Simonsohn et al., 2014). We found that for most of the parameters, there were no substantial publication biases in these datasets (Supplementary Table 2). For some parameters which showed some biases, then we took the effect sizes (SMD), CIs, and heterogeneity statistics after applying the trim-and-fill method to correct the biases (Duval and Tweedie, 2000). However, for the majority of the cases, the trim and fill did not substantially alter (decreased) the SMD values than the untrimmed SMD values (Supplementary Table 2). Therefore, we used the untrimmed SMD values (i.e., did not adjust for publication bias) for the sake of consistency for all the parameters for creating forest plots. After carefully observing the heterogeneity statistics as well as significance level for SMDs, we performed subgroup analyses for root length parameters to determine the influence of the factors such as plant or endophyte identity, type, etc., because sufficient data were available. For a factor to be included in the analysis as a subgroup variable, it had to be reported from at least four studies.

## Meta-Analytic Review and Discussion on Plant Tolerance to Thermal Stress as Mediated by Microbial Colonization

**FIGURE 1:**
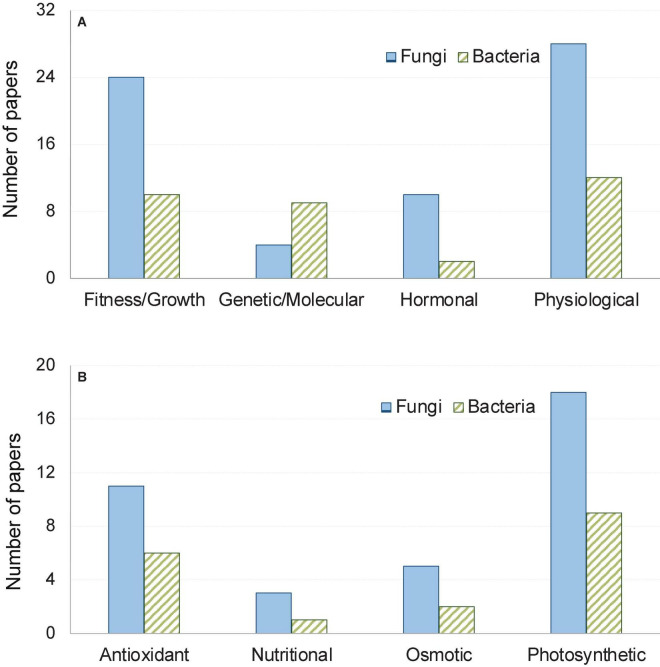
Number of papers out of the total 42 papers reviewed here grouped by response variables. Broad categories of variable are shown in panel **(A)**, while physiological responses are shown in panel **(B)**.

The articles we reviewed presented findings of experiments involving various host-microbe systems. These investigations considered various parameters in evaluating the influences of microbial colonization in providing heat stress tolerances of plants such as plant biomass and response at the physiological, molecular, metabolic as well as the hormonal level (Figure 1). Effect of fungi was encountered more often, which accounted for 69% of the 42 articles used in our meta-analysis. In general, both fungal and bacterial symbionts were found to increase plant fitness under thermal stress by augmenting photosynthesis, improving antioxidant responses of plants, triggering earlier hormonal signaling, enhancing osmolyte and nutritional balance (Table 1). In the next section, we outlined the influence of microbial symbionts on plant thermal stress tolerance as obtained from our meta-analysis and discussed in light of available literature.

**TABLE 1:** The 39 investigations that were included in the review here reporting the effects of microbial symbionts on plant responses to thermal stresses.

References	Host species	Symbiont	Key findings
Abd El-Daim et al. (2014)	Wheat (*T. aestivum*)	*Bacillus amyloliquefaciens; Azospirillum brasilense* (Bacteria)	Bacterial treatment with *Bacillus amyloliquefaciens* UCMB5113 or *Azospirillum brasilense* NO40 improved heat stress tolerance of wheat seedlings which were associated with reduced generation of reactive oxygen species and transcript levels of several stress related genes.

Abd El-Daim et al. (2018)	Wheat (*Triticum aestivum*)	*B. amyloliquefaciens* (Bacteria)	Wheat seeds treated with *B. amyloliquefaciens* enhanced heat tolerance by molecular modifications in wheat leaf transcript patterns.

Abd El-Daim et al. (2019)	Wheat (*T. aestivum*)	*B. velezensis* (Bacteria)	*B. velezensis* 5113 inoculations resulted in a significant metabolic modulation and regulation of metabolic pathways of amino acids and enhanced expression of several proteins related to heat tolerance.

Ali et al. (2009)	*Sorghum (Sorghum bicolor)*	*Pseudomonas sp.* (Bacteria)	*Pseudomonas* sp. strain AKM-P6 enhanced tolerance of sorghum seedlings to elevated temperatures by enhancing bio-physiological changes such as higher biomass, proline, sugar, amino acids content in the plant.

Ali et al. (2011)	Wheat (*Triticum spp*.)	*P. putida* (Bacteria)	Inoculation with *P. putida* strain AKMP7 significantly increased the root and shoot length, dry biomass, tiller, spikelet and grain formation of wheat and enhanced biochemical parameters such as chlorophyll, total sugars, proline, starch, amino acids and protein content of wheat under heat stress.

Ali et al. (2018)	Cucumber (*Cucumis sativus*)	Theromophilic endophytic fungus	A thermophilic endophytic (CpE) fungus (92% sequence homology with *Thermomyces sp*.) mediated heat stress tolerance in cucumber plants by maintaining maximum quantum efficiency of photosystem II, photosynthesis rate, water use efficiency and increased root length.

Ali et al. (2019)	*Cullen plicata*	*Thermomyces lanuginosus* (Endophytic fungus)	*T. lanuginosus* had a beneficial effect on its host for resisting heat under heat stress condition. AM fungus enhanced the *C. plicata* plant heat stress tolerance by changing secondary metabolite accumulations and antioxidant activities and improving the growth and development of its host.

Bruno et al. (2020)	*Sorghum (S. bicolor)*	*B. cereus, Providencia rettgeri, Myroides odoratimimus* (Bacteria)	Inoculation of sorghum with chromium reducing-thermotolerant plant growth promoting bacteria (CRT-PGPB) increased plant growth, antioxidant enzyme activities and decreased proline and malondialdehyde contents in plants under heat stress and enhanced heat tolerance

Bunn et al. (2009)	*Agrostis scabra, Dichanthelium lanuginosum, Mimulus guttatus*	Arbuscular mycorrhizal fungi	Biomass of the facultative thermal plants *Agrostis scabra* and *Mimulus guttatus* decreased under high temperature and AMF inoculation did not have any effect on the plant traits. On the other hand, mycorrhizal inoculation increased the biomass and total root length of the obligate thermal plant *Dichanthelium lanuginosum* under high temperatures. Again, the source of the AMF inoculum (nonthermal-AMF and thermal-AMF), had no effect on colonization level, host plant biomass, or flowering for any host species in either temperature treatment, suggesting that AMF from thermal soils are not specifically adapted to higher temperatures.

Cabral et al. (2016)	Wheat (*T. aestivum)*	Arbuscular mycorrhizal fungi	AMF increased grain number in wheat plants, alter nutrient allocation and tiller number composition in the plants under heat-stress.

Duc et al. (2018)	Tomato (*Solanum lycopersicum*)	*Septoglomus deserticola and Septoglomus constrictum* (Arbuscular mycorrhizal fungi)	Inoculation with AMF reduced oxidative stress by decreasing lipid peroxidation and hydrogen peroxide levels and enhancing antioxidant enzyme activities.

Eerens et al. (1998)	Ryegrass (*Lolium perenne*)	*Neotyphodium lolii* (Endophytic fungus)	The endophyte affects the morphology and physiology of the ryegrass plant. The interaction between endophyte and temperature was significant for ergovaline concentration and wilt score.

Hennessy et al. (2016)	Perennial ryegrass (*L. perenne* L.); Italian ryegrass (*L. multiflorum*Lam.)	*Epichlo festucae* (Endophytic fungus)	The endophyte strain AR37 (*Epichlo festucae* var. lolii) provided ryegrass with resistance against *Porina larvae*, a major pasture pest in cooler areas of New Zealand by producing high concentrations of epoxy-janthitrem alkaloids at high temperature. In contrast, AR37-infected ryegrass grown at low temperature produced low concentration of epoxy-janthitrem resulting in a small anti-feedant effect against pasture pest only in perennial ryegrass.

Hubbard et al. (2012)	Durum wheat (*T. turgidum)*	Endophytic Ascomycetous mitosporic fungi	Fungal endophytes increased wheat seed germination percentage, improved the hydrothermal time (HTT) and energy of germination (EG) value and enhanced resistance to wheat exposed to heat *in vitro* as measured by fresh weight of seedlings
Hubbard et al. (2014)	Durum wheat (*T. turgidum*)	Endophytic Ascomycetous mitosporic fungi	Endophytes enhanced wheat tolerance against heat stress in parental plants and second-generation seeds which was measured by quantifying efficiency of photosystem II, average seed weight (ASW), total seed weight (TSW), water-use efficiency (WUE), time to 50% germination and percentage germination of second-generation seeds produced under heat stress.

Ismail et al. (2018)	Sunflower (*H. annuus*); Soybean (*G. max*)	*A. japonicus* (Endophytic fungus)	*A. japonicus* EuR-26 endophytic fungus isolated from the wild plant *Euphorbia indica* L. have heat stress relief potential. *A. Japonicus* can modulate the growth of host plants under heat stress and can be used in arid and semiarid regions of the world as a thermal stress alleviator.

Ismail et al. (2019)	Sunflower (*H. annuus*) and Soybean (*G. max*	*A. flavus* (Endophytic fungus)	Harms due to heat stress on soybean and sunflower appeased by inoculating endophytic fungi *A. flavus* isolated from *Euphorbia indica*. In fact, the endophytic fungi influenced to regulate the concentration of ABA, proline, phenols, flavonoids, catalase and ascorbic acid oxidase to withstand soybean and sunflower plants against heat stress.

Ismail et al. (2020a)	Sunflower (*Helianthus annuus*); Soybean (*Glycine max*)	*A. niger* (Endophytic fungus)	Endophytic fungi *A. niger* isolated from *Sonchus asper*protected soybean and sunflower from excessive thermal stress by increasing plant height, biomass and chlorophyll content. In fact, *A. niger* alter plant physiology in such a way that, concentration of AAO, CAT, GR, SOD, POD, proline and phenolics were augmented while lipid peroxidation, ROS ad ABA reduced under thermal stress at 40°C.

Ismail et al. (2020b)	Sunflower (*H. annuus*); Soybean (*G. max*)	*A. violaceofuscus* (Endophytic fungus)	Endophytic fungi *A. violaceofuscus* isolated from the fern *Dryopteris flix* L. protected sunflower and soybean against thermal stress through secreting secondary metabolites. Such secretion increased the total chlorophyll content, plant height by decreasing the concentration of reactive oxygen species, abscisic acid, catalase, ascorbic acid oxidase, proline.

Ismail et al. (2020c)	Sunflower (*H. annuus*); Soybean (*G. max*)	*Rhizopus oryzae* (Endophytic fungus)	Thermal stress to soybean and sunflower caused by climatic change can be mitigated to a great extent by using *R. oryzae*, an endophytic fungus isolated from roots and leaves of *Adiantum capillus-veneris* L. The plant growth promoting fungus, *R. oryzae*, improved heat stress tolerance of plants through inducing high concentration of phenolics, flavonoids, salicylic acid (SA) and indole 3-acetic acid (IAA), ascorbic acid oxidase (AAO), catalase (CAT), proline, sugars, proteins and lipids.

Issa et al. (2018)	Tomato (*S. lycopersicum*)	*Paraburkholderia phytofirmans* (Bacteria)	Bacterial inoculation with *P. phytofirmans* strain PsJN improved tomato plant growth by enhancing chlorophyll content and gas exchange parameters and increased the accumulations of sugars, total amino acids, proline, and malate.

Khan et al. (2012a)	*C. sativus*	*Paecilomyces formosus* LHL10 (Endophytic fungus)	The association with endophytic fungus, *P. formosus* LHL10 significantly improved growth parameters of cucumber plants such as higher leaf area, chlorophyll contents, shoot fresh and dry weights compared to control even under stress conditions. *P. formosus* helped the plant to cope with the adverse effects of temperature stress, especially low-temperature stress.

Khan et al. (2012b)	*C. sativus*	*Exophiala sp. LHL08* (Endophytic fungus)	*Exophiala* sp. inoculated plants had significantly higher plant growth characteristics (shoot length, plant biomass, chlorophyll quality, and leaf area) than heat stress control. Exophiala sp.’s interaction with cucumber host plants can modulate heat stress by influencing plant physiological and biochemical traits under heat stress.

Khan et al. (2013)	Pepper (*Capsicum annuum)*	*Penicillium resedanum* (Endophytic fungus)	The endophytic fungus *P. resedanum* LK6 significantly increased the shoot length, shoot fresh and dry wrights of *C. annuum* L. plant with or without the exposure to heat stress as compared to non-infected plants. The leaf damage and wilting process were significantly lower in endophyte-treated plants than control under heat stress. Proline and ammonium accumulation and flavonoids synthesis were significantly induced in the endophyte-inoculated plants.

Khan A. L. et al. (2015)	Pepper (*C. annuum*)	*P. resedanum* (Endophytic fungus)	*P. resedanum* LK6 treatment significantly improved shoot length and shoot dry weight of capsicum plants compared with the controls under heat stresses. Endophyte helps the host to response against stresses by reprogramming the physiological responses of host plant.
Khan et al. (2020a)	Tomato (*S. lycopersicum*)	*B. cereus* (Bacteria)	Simultaneous use of plant growth-promoting endophytic bacteria *B. cerus* and humic acid (HA) can counteract the negative effects of heat stress. Co-application of *B. cereus* SA1 and humic acid (HA) on tomato seedlings can protect against heat stress by balancing required biochemicals like ABA, SA, APX, GSH, SOD, Fe, P, K. As a result, SA1 and HA can be used as a commercial biofertilizer.

Khan et al. (2020b)	Soybean (*G. max*)	*B. cereus* (Bacteria)	Negative impact of heat stress on growth and yield of soybean due to global climatic change can be mitigate by using thermo tolerant *B. cereus* bacteria. In fact, *B. cereus* can be used as a bio fertilizer as it can promotes biologically active metabolites like GA, IAA and organic acids besides maintaining optimum balance of ABA, SA, protein ad antioxidant concentration.

Li X. et al. (2021)	Ryegrass (*L. perenne*)	*A. aculeatus* (Endophytic fungus)	Inoculation with fungus improved heat tolerance by enhancing the photosynthetic apparatus, decreased the antioxidant enzyme activities, and mitigated membrane lipid peroxidation.

Martin and Stutz (2004)	Pepper (*C. annuum*)	*Glomus intraradices, Glomus sp. (AZ 112)* (AM fungi)	At moderate temperatures, phosphorus uptake by all AM colonized pepper plants was enhanced relative to non-AM plants but there was no corresponding enhancement of growth. In contrast, at high temperature, pepper growth was increased as a result of inoculation of the *G. intraradices* isolate and the Glomus isolate mixture as compared to non-AM controls, despite relatively less phosphorus transfer to the plant by the fungi.

Mathur and Jajoo (2020)	Maize (*Zea mays*)	*Glomus sp.*	AMF exerted their beneficial effects by altering PSII heterogeneity under high temperature. Presence of AMF was able to protect maize plants by regulating electron transport through PSII and thus regulating reducing side heterogeneity of PSII under high temperature stress.

Maya and Matsubara (2013)	*Cyclamen persicum*	*G. fasciculatum (AM fungus)*	Symbiotic association of *G. fasciculatum*, an arbuscular mycorrhizal (AM) fungus, and *Cyclamen persicum* mitigated heat stress damage by enhancing antioxidant concentration like superoxide dismutase, ascorbate peroxidase, ascorbic acid, and polyphenol. Moreover, compare to control mycorrhizal symbiosis provokes higher plant biomass in the host plants.

Mukhtar et al. (2020)	Tomato (*S. lycopersicum*)	*B. cereus* (Bacteria)	Bacterial inoculation promoted shoot, root length, leaf surface area, fresh and dry weight and enhanced extracellular polymeric substances (EPS) production and reduced the adverse effects of heat on tomato growth.

Sangamesh et al. (2018)	Rice (*Oryza sativa*)	*Chaetomium sp.* (Fungus)	Inoculation with *Chaetomium* sp. increased root and shoot growth as well as survival percentage under heat stress.

Sarkar et al. (2018)	Wheat (*T. aestivum*)	*B. safensis and Ochrobactrum pseudogrignonense* (Bacteria)	PGPR enhanced thermotolerance by reduction of ROS production, membrane damage, maintenance of chloroplast structure and enhanced chlorophyll content, increased expression of an array of redox enzymes and accumulation of osmolytes.

Shekhawat et al. (2020)	*Arabidopsis thaliana*	*Enterobacter sp.* (Endophytic bacterium)	*Enterobacter* sp. SA187 increased significant heat stress tolerance in *A. thaliana*. *Enterobacter* sp. SA187 enhanced the expression of heat-responsive and heat memory-related genes upon heat stress, therefore helping plants to survive better to heat stress.

Tian et al. (2015)	*Festuca rubra*	*Epichlo festucae* (Endophytic fungus)	*E. festucae* infection did not affect the physiological responses of the plants on improving heat stress tolerance in strong creeping red fescue.

Waqas et al. (2015)	Rice (*O. sativa*)	*P. formosus* (Endophytic fungus)	The phytohormones and other secondary metabolites formed by the endophytic fungus *P. formosus* LWL1 in the Dongjin Japanese rice cultivar played a potential role in heat-stress mitigation. These fungal endophytes may be useful for the sustainable production of crops under high ambient temperature levels as they showed positive result.

Yeasmin et al. (2019)	*Asparagus officinalis*	*G. intraradices (AM fungus)*	Arbuscular mycorrhizal fungi (AMF) *G. intraradices* ameliorated damage of asparagus crop caused by heat stress. AMF-inoculated asparagus plants compare to control performed better regarding growth, nutrient uptake, heat stress responses. Besides, increased antioxidative activities of SOD and ascorbate peroxidase in inoculated plants lessen leaf browning.
Zhu et al. (2011)	Maize (*Z. mays*)	*G. etunicatum (AM fungus)*	Compared to control, maize plants inoculated by AM fungus *G. etunicatum* showed better performance regarding photosynthesis, stomatal conductance, and transpiration in leaves. Furthermore, AM fungus may protect maize plants against high temperature stress by improving water use efficiency.

### Microbial Symbionts on Plant Growth and Relative Water Content Under Thermal Stress

**FIGURE 2:**
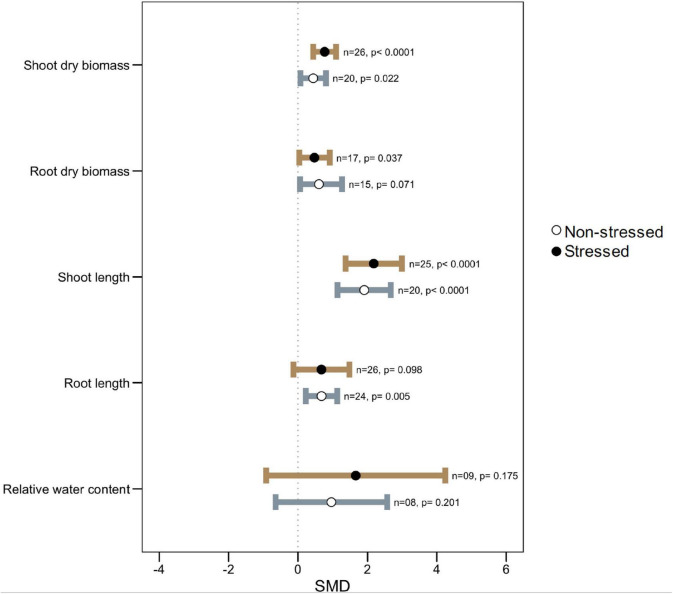
Growth responses of microbe-inoculated plants compared with those of non-inoculated plants under heat stress and non-stressed conditions. Error bars are effect size (SMD) means ± 95% CIs. Where the CIs do not overlap the vertical dashed lines, the effect size for a parameter is significant, i.e., the growth responses of inoculated plants were significantly different from those of non-inoculated plants. *n* = number of studies included in the meta-analysis, *p* = significance level of SMD.

The microbial symbiosis significantly stimulated plant shoot dry biomass as well as increased shoot length both under normal and high-temperature stress (Figure 2). Although root dry biomass was found to be significantly augmented (SMD: 0.4728, *p:* 0.037) by microbial colonization under heat stress, its effect on the length of the root was variable and less prominent (SMD: 0.6739, *p:* 0.098) even though under non-stressed conditions microbial colonization resulted in longer roots in host plants (Figure 2).

Leaf relative water content (RWC) did not show any significant changes due to microbial colonization either under stress or non-stressed conditions. However, the wider CI values propose that the influence on plant RWC varies by some factors (Figure 2).

**FIGURE 3:**
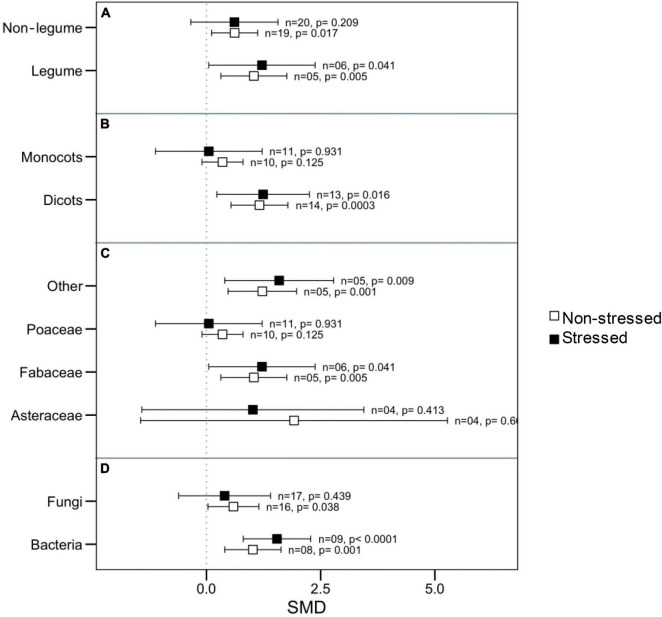
Effects of microbial inoculation on plant root length under normal and high temperature stress conditions for various categorical variables such as **(A)** Plant nodulation, **(B)** Plant clade, **(C)** Plant Family, and **(D)** microbes’ types (fungi or bacteria). Error bars are the effect size means ± 95% CIs. Where the CIs do not overlap the vertical dashed lines, the effect size for a parameter is significant, i.e., the growth responses of AMF plants were significantly different from those of non-AMF plants. *n* = number of studies included in the meta-analysis, *p* = significance level of SMD.

Since there was significant heterogeneity in microbial impact on plant root length, we attempted to identify the potential moderators by categorical analysis. We noticed that microbes had a substantial impact on root length regardless of whether the host belonged to the legume with non-legume or whether the symbiont belonged to fungi or bacteria under thermal stress (Figures 3A,D). However, under normal conditions, bacterial symbiont had significantly increased root length, but fungi did not demonstrate this effect. The roots of dicotyledonous plants increased significantly in the presence of microbial symbionts regardless of stress impact, but those of monocotyledons did not show this response (Figure 3B). When considering the plant family as a moderator, Poaceae and Asteraceae had an overall neutral influence in root length, but Fabaceae and those belong the other family had significantly positive responses to microbial colonization under both stressed and normal temperature (Figure 3C).

### Microbial Symbiosis on Plant Photosynthesis Under Thermal Stress

**FIGURE 4:**
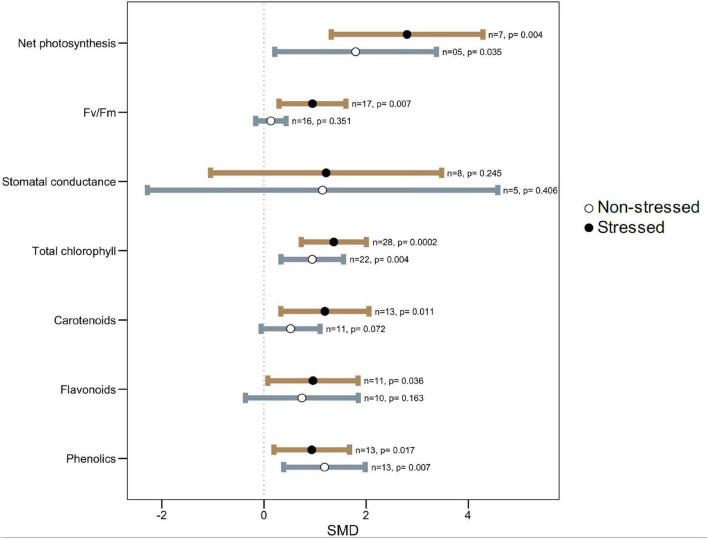
Effect of microbe inoculation on photosynthetic parameters compared with those of non-inoculated plants under heat stress and non-stressed conditions. Error bars are effect size (SMD) means ± 95% CIs. Where the CIs do not overlap the vertical dashed lines, the effect size for a parameter is significant, i.e., the growth responses of inoculated plants were significantly different from those of non-inoculated plants. *n* = number of studies included in the meta-analysis, *p* = significance level of SMD.

All photosynthetic parameters considered, however, tended to be influenced more under stress than under non-stress conditions, as evident from their larger effect sizes (Figure 4). Microbial colonization significantly increased net photosynthesis, maximum photochemical efficiency (Fv/Fm), and photosynthetic pigments such as chlorophyll, carotenoids, flavonoids, and phenolics in plants under high-temperature stress as compared to non-colonized plants. However, under normal temperature, these effects were not as strong as under stressed conditions (Figure 4). For example, symbiotic plants had 25% (1.37, *p* = 0.0002) and 51% (2.80, *p* = 0.004) higher total chlorophyll and net photosynthesis, respectively, over non-symbiotic plants under stress but these were only 11% (0.94, *p* = 0.004) and 16% (1.79, *p* = 0.035) higher under ambient conditions (Figure 4). There was no significant effect on stomatal conductance in general, but the wider CI-values, as well as heterogeneity statistics, showed that it varies due to different plant-microbe systems.

Improvement in the photosynthetic ability of symbiotic plants is correlated clearly with the increased plant biomass of inoculated plants. Thermal stress exerts a negative impact on photosynthetic machinery and gas exchange in plants causing altered physiological and biochemical processes. Similar to heat stress, early reaction to water deficit could result in accelerated stomatal closure and reduced water loss (Malinowski and Belesky, 2000) in microbial colonized plants. Also, an augmented chlorophyll pigment in symbiotic plants under stress reflects their better photosynthesis which corelates with their stress tolerance (Harman, 2000; Harman et al., 2004; Bae et al., 2009). Carotenoids and flavonoids are efficient quenchers of ROS, such as peroxide radicals and singlet oxygen molecules, and thus abate oxidative damage (Wahid et al., 2007; Agati et al., 2012). Increased carotenoids content in microbe-treated plants compared to untreated under heat stress provides evidence of involvement of this antioxidant in the microbe mediated ROS scavenging pathway for increasing plant heat stress tolerance (Figure 4). It has been shown that carotenoids of the xanthophyll family and some other terpenoids bring about a decreased fluidity (thermostability) of the lipid membrane, resulting in reduced susceptibility to lipid peroxidation under high temperatures (Havaux, 1998; Velikova et al., 2004; Sharkey, 2005). It is also worthwhile mentioning that in a recent paper it was demonstrated that plants grown under warmer conditions are able to better control oxidative stress and lipid peroxidation thanks to the activation of higher antioxidant defenses (Bertini et al., 2021). Phenolics, flavonoids, anthocyanins, lignins, etc., are important secondary metabolites in plants and are involved in stress tolerance in the plant (Chalker-Scott, 2002; Wahid, 2007; Wahid et al., 2007). We noted that microbial colonization significantly increased phenolics and flavonoids in plants under heat stress (Figure 4). Increased accumulation of phenolics under heat stress was shown to be associated with higher phenyl ammonia-lyase (PAL) and lower peroxidase and polyphenol lyase activities (Rivero et al., 2001).

### Microbial Symbiosis on Osmolytes and Hormones Accumulation Under Thermal Stress

One of the important mechanisms plants employ to withstand various stresses, including extreme temperatures stress, is the accumulation of certain organic compounds known as compatible osmolytes (Hare et al., 1998; Sakamoto and Murata, 2002). Depending on the plant species and stress type and severity, various compounds such as sugars, sugar alcohols, proline, glycinebetaine (GB), and tertiary sulfonium compounds are accumulated (Sairam and Tyagi, 2004). In plants, GB plays a vital role as an osmolyte under various stresses, such as drought, salinity, or high temperature (Giri, 2011). Different plants showed varying capacity to synthesize GB under stress (Ashraf and Foolad, 2007; Wahid et al., 2007). However, we did not come across any study that investigated the involvement of GB in microbial-mediated plant heat stress tolerance; there is room for future researchers to find out the role of this compound.

**FIGURE 5:**
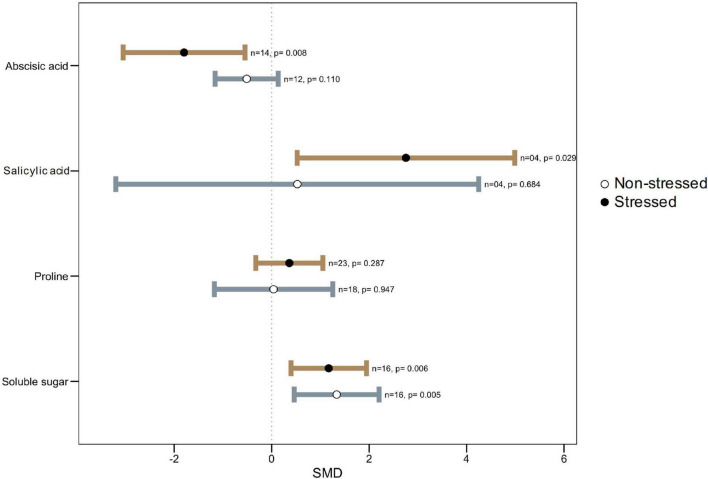
Effect of microbe inoculation on osmplytes and hormones compared with those of non-inoculated plants under heat stress and non-stressed conditions. Error bars are effect size (SMD) means ± 95% CIs. Where the CIs do not overlap the vertical dashed lines, the effect size for a parameter is significant, i.e., the growth responses of inoculated plants were significantly different from those of non-inoculated plants. *n* = number of studies included in the meta-analysis, *p* = significance level of SMD.

Similar to GB, proline also accumulates in plants under stresses and take part in plant abiotic stress tolerances. Proline accumulation helps in osmotic adjustment and plays a protective role as a ROS scavenger under thermal stress (Szabados and Savouré, 2010; Kaur and Asthir, 2015). However, it is still controversial if its presence is an adaptive response that provides greater stress tolerance or if its increase is a symptom of stress injury (Ashraf and Foolad, 2007). Apparently, no significant difference in proline accumulation in colonized plants versus non-colonized plants (Figure 5) suggests that the effect of proline is not independent but relies on many factors, one among them being phytohormones (Iqbal et al., 2019). Similar findings were reported on proline accumulation and its association in microbe-mediated plant drought tolerance (Dastogeer, 2018). It was hypothesized that relatively higher levels of proline in microbial colonized plants indicate a reduced extent of damage in a stressed plant (Dastogeer and Wylie, 2017; Dastogeer et al., 2018). Proline functions as low-molecular weight chaperones in plants and helps avoid the stress effect (Gupta et al., 2013). Because of its zwitter ion nature, proline accumulates to high-concentration in cell cytoplasm during stress. Yet, over-accumulation of proline could show toxicity to plant cells (Rizhsky et al., 2004). Genotypic variations for varying levels of proline due to stress have been known in earlier studies with sunflower (Amutha et al., 2007; Harsh et al., 2016). Therefore, the status of proline level is not a reliable indicator of stress tolerance of plants (Silvente et al., 2012).

Similarly, the accumulation of soluble sugars under heat stress has been reported in sugarcane, which entails great implications for heat tolerance (Wahid and Close, 2007). Under high temperatures, fruit set in tomato plants failed due to the disruption of sugar metabolism and proline transport during the narrow window of male reproductive development (Sato et al., 2006). Among other osmolytes, 4-aminobutyric acid (GABA), a non-protein amino acid, is widely distributed throughout the biological world to act as a compatible solute. Several studies showed that various environmental stresses increase GABA accumulation through metabolic or mechanical disruptions, thus leading to cytosolic acidification. Rapid accumulation of GABA in stressed tissues may provide a critical link in the chain of events stemming from perception of environmental stresses to timely physiological responses (Kinnersley and Turano, 2000).

Phytohormones, like abscisic acid (ABA), salicylic acid (SA), and jasmonic acid (JA), serve as signaling compounds during stress (Shinozaki and Yamaguchi-Shinozaki, 2007). ABA, an isoprenoid phytohormone, is involved in the regulation of many physiological processes in plants, including stomatal opening and closing, protein storage, and adaptation to stress. In nature, where heat and drought stress usually coincide, induction of ABA is an important element of thermotolerance, pointing to its role in biochemical pathways essential for survival under heat stress (Maestri et al., 2002; Sah et al., 2016). Several studies highlighted that induction of various heat shock proteins (HSPs) (e.g., HSP70) by ABA may be one mechanism whereby it confers thermotolerance (Pareek et al., 1998). Interestingly, however, in our meta-analysis, ABA level was found significantly lower in colonized plants as compared to the non-colonized plants (Figure 5). Some microbes produce biologically active gibberellins (GAs), which might be associated with plant growth promotion through reducing stress hormones like ABA (Zhang et al., 2014; Verma et al., 2016; Bashar et al., 2019). Under stressful conditions, the plant regulates stress hormones like ABA through active chemical signals, which induce extreme sensitivity to stomatal conductance (de Ollas and Dodd, 2016; Verma et al., 2016).

The salicyclic acid (SA) is an important signaling component in response to systemic acquired resistance (SAR) and the hypersensitive response (HR) (Kawano et al., 1998) and modulates plant growth, development, flowering, stomatal response, ethylene synthesis, and respiration (Waqas et al., 2012; Khan M. I. R. et al., 2015; Wani et al., 2017). Various studies highlighted the roles of SA in improving plant tolerance to heat stress (Larkindale and Knight, 2002; Wang L.-J. et al., 2010; Khan M. I. R. et al., 2015). The SA stabilizes the trimers of heat shock transcription factors and helps them bind heat shock elements to the promoter of heat shock-related genes. SA-induced thermotolerance involves Ca^2+^ homeostasis and antioxidant systems (Wang and Li, 2006). Our meta-analysis showed that microbial inoculation greatly enhanced SA levels in plants under heat stress conditions (Figure 5).

The Jasmonic acid (JA) has roles in the biosynthesis of defensive secondary metabolites and proteins (Balbi and Devoto, 2008) as well as it is involved in many physiological processes such as resistance to insects and pathogen, pollen development, senescence, and root growth (Lorenzo et al., 2004). Khan et al. (2012b) and Waqas et al. (2015) reported a substantially low induction of this hormone in microbial colonized plants under stress suggesting its involvement in heat tolerance. The JA level was increased in *Exophiala* sp.-inoculated plants under growth conditions significantly reduced as compared to non-inoculated plants (Waqas et al., 2015). Further studies are needed to observe if reduction of JA is common in all microbe-mediated thermo-tolerance and what are the underlying mechanisms involved.

Another hormone, ethylene is involved in almost all growth and developmental processes in plants, including stress tolerance (Iqbal et al., 2017). Variable responses in ethylene production (i.e., increase or decrease) in plants have been reported under heat stress conditions (Antunes and Sfakiotakis, 2000; Arshad and Frankenberger, 2002; Larkindale and Huang, 2005). We did not come across any study that looked into ethylene production for microbial colonized versus non-colonized plants under normal or stress conditions, and this is an area of future research.

The hormones gibberellins and cytokinins show contrasting roles on plant heat tolerance to ABA (Liu and Hou, 2018; Li N. et al., 2021). However, there are insufficient studies on the effect of gibberellins and cytokinins on microbial conferred heat tolerance for inclusion into our meta-analysis. In a study, an endophyte *Penicillium resedanum* LK6 produces gibberellins and improves growth, and provides resilience to environmental stresses (Khan A. L. et al., 2015). Further investigation is necessary for revealing the involvement of gibberellins and cytokinins in this regard.

### Microbial Symbiosis on Activity of Antioxidant Enzymes Under Thermal Stress

Reactive oxygen species (ROS) are produced in the plant as by-products of aerobic metabolism and exist in several forms such as hydroxyl radicals (^•^OH) and superoxide anions (O_2_^–^), hydrogen peroxide (H_2_O_2_), or singlet oxygen (^1^O_2_) (Blokhina et al., 2003; Apel and Hirt, 2004; Mittler et al., 2004). The ROS functions as signaling molecules, at low levels, they are necessary for basic biological processes of the plant, but higher levels are detrimental and may cause DNA damage and incorrect timing of programmed cell death (PCD) directly (Tsukagoshi et al., 2010; Zafra et al., 2010; Xie et al., 2014). The plant has an efficient well-coordinated, and rapidly responsive antioxidant system, including both non-enzymatic and enzymatic processes to mitigate the effect of overproduction of ROS. However, the equilibrium between synthesis and removal of ROS may be perplexed under various biotic and abiotic stresses (Quan et al., 2008; Huang et al., 2019). For example, at higher temperatures, there is an increased production of ROS causing a disparity between ROS production and the ability of the scavenging process to detoxify and remove the reactive intermediates (Saidi et al., 2011; Hemantaranjan et al., 2014). The association of microbial symbiosis has been described for higher tolerance to different environmental stresses (Hernandez et al., 2000; Larkindale and Knight, 2002; Halliwell, 2006). Thus, the involvement of the microbiome in mitigating oxidative stress in the plant has recently attracted research interest.

### Ascorbate-Glutathione Cycle

**FIGURE 6:**
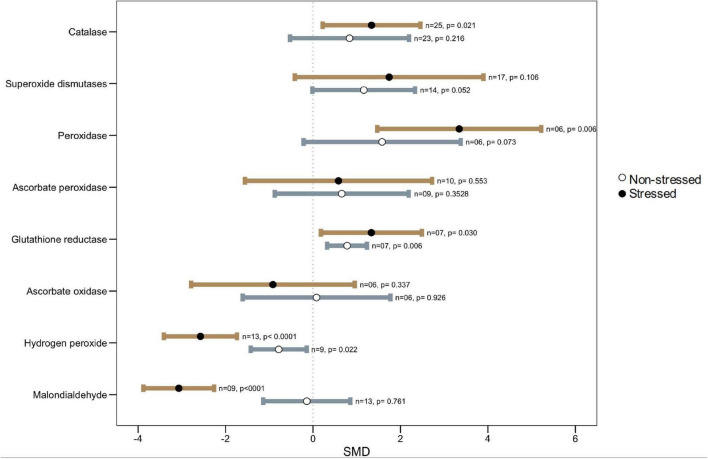
Effects of microbial inoculation on plant ROS production and antioxidant enzymatic activity under non-stressed, and heat stress. Error bars are means ± 95% CIs. Where the CIs do not overlap the vertical dashed lines, the effect size for a parameter is significant. *n* = number of studies included in the meta-analysis, *p* = significance level of SMD.

It has been shown in several studies that microbial-mediated plant heat stress tolerance is associated with heightened antioxidant defense in inoculated plants. The contributions of the ascorbate-glutathione (AsA-GSH) cycle on plant tolerance under various abiotic stresses have been reported in many plant species (Anjum et al., 2010; Bartoli et al., 2017; Kuźniak et al., 2017). The four enzymes that steer the cycle are ascorbate peroxidase (APX), dehydroascorbate reductase (DHAR), mono-dehydroascorbate reductase (MDHAR), and glutathione reductase (GR) (Quan et al., 2008; Noctor et al., 2014; Mohi-Ud-Din et al., 2021). In our analysis, we found that there were no overall changes in APX activity in colonized plants under heat stress (Figure 6). However, the wider CI values indicated that the APX accumulation is variable depending on host-microbe types or other factors. For example, it was reported that treatment with bacteria (*Bacillus amyloliquefaciens* or *Azospirillum brasilense*) decreased the activity of this enzyme as well as significantly lower expression of the APX1 gene was observed in heat exposed wheat seedlings (Abd El-Daim et al., 2014). A strikingly different finding was reported in several other studies where microbial treatment increased the level of APX accumulation over non-treated plants under high heat conditions (Maya and Matsubara, 2013; Yeasmin et al., 2019; Khan et al., 2020b). The other two enzymes: DHAR and MDHAR, could not be included in our meta-analysis because of the insufficient number of reports, but both of them were found to increase significantly in plants under elevated temperature, and microbial colonization results in lower accumulation as compared to non-colonized plants (Abd El-Daim et al., 2014, 2019). In general, a significantly higher accumulation of GR in the microbial colonized plants over non-colonized plants was observed through our meta-analysis (Figure 6). The GR plays a crucial role in controlling the levels of reduced glutathione in the plant (Huang et al., 2019). Glutathione modulates ROS levels in cells through both auxin/PLETHORA (PLT) dependent and independent pathways, thereby participating in and maintaining ROS homeostasis in the plant (Yu et al., 2013).

#### Antioxidant Enzymes

Apart from the AsA-GSH cycle, SOD (superoxide dismutase), CAT (catalase), POD (peroxidase), glutathione peroxidases (GPX), and glutathione-*S*-transferases (GST) are important antioxidant enzymes in plants taking part in ROS homeostasis under stress. In the plant cells, SOD provides primary protection against O_2_^•–^, which is then converted to H_2_O_2_ for subsequent metabolism to H_2_O by CAT and peroxidases (POD, APX, and GPX), thus protecting the cell damage (Mittler et al., 2004; Gill and Tuteja, 2010; Wang G. et al., 2010; Mohi-Ud-Din et al., 2021). The current meta-analysis showed no apparent disparity in SOD measurement in colonized over non-colonized plants, but the higher CI values indicated that SOD accumulation varied significantly among studies. For example, in a study, Li X. et al. (2021) did not notice any changes in SOD accumulation in *Aspergillus aculeatus* inoculated *Lolium perenne*, although this fungus improved the heat tolerance of this plant. Similar results have been reported for *Septoglomus deserticola* and *Septoglomus constrictum* (AM fungi) mediated heat stress tolerance in the tomato plant with no apparent changes in SOD activity (Duc et al., 2018). In contrast, a few studies reported a significant increase in SOD in microbial colonized plants under high-temperature stress (Maya and Matsubara, 2013; Yeasmin et al., 2019; Bruno et al., 2020; Khan et al., 2020a). Regarding CAT and POD, they showed consistently higher accumulation (CAT, *p* = *0.021*; POD, *p* = *0.006*) in colonized plants as compared to non-colonized controls under heat stress across various studies (Figure 6).

Also, a significantly decreased level of hydrogen peroxide (H_2_O_2_) and Malondialdehyde (MDA) (*p* < 0.001) in microbial-treated plants adds further support to a lower level of heat stress injury due to the increased induction of the antioxidant defense systems compared to the non-treated plants (Figure 6). The MDA is the principal and extensively studied product of polyunsaturated fatty acid (PUFAs) peroxidation. The MDA levels are used as a measure of membrane lipid peroxidation and oxidative stress and the impact it has on membrane fluidity, leakiness, and damage of membrane proteins, enzymes, and ion channels (Garg and Manchanda, 2009; de Dios Alché, 2019).

## Microbial Symbiosis on Plant Thermotolerance: Molecular Aspects

There is a huge lack of understanding of the mechanisms of microbe-mediated plant heat stress tolerance at the molecular level. The heat shock protein (HSPs) act as “molecular chaperones,” and their upregulation is well known to increase thermotolerance in plants. The expression of heat stress-dependent regulatory proteins such as heat shock transcription factors (HSFs) and heat shock proteins (HSPs) have been described. The HSPs are crucial in regulating and restoring protein structures and maintaining the condition of plants under heat stress (Queitsch et al., 2000; Nakamoto and Vigh, 2007; Xu et al., 2012; Wang et al., 2014). Several studies reported an increased level of expression of HSPs in the microbial colonized plant compared to non-colonized plants under heat stress (Abd El-Daim et al., 2019; Bruno et al., 2020; Li X. et al., 2021). To reveal the mechanisms through which Enterobacter sp. SA187 mediates plant thermotolerance in *Arabidopsis* Shekhawat et al. (2020) carried out RNA-seq and targeted transcriptomic analyses. They described that the bacteria-mediated heat tolerance is associated with the gene networks for heat protection of plants similar to that of the heat priming (pre-exposure of plants to heat that enables plants to be better prepared to response to heat stress). For example, heat tolerance is associated with the upregulation of genes related to the auxin and gibberellin hormones and the down-regulation of the genes related to jasmonic and abscisic acid. It was also found to be related to hyper-induction of heat-responsive and heat memory genes, which is proven in heat primed plants (Lämke et al., 2016; Ling et al., 2018; Liu et al., 2018). The bacteria inoculated plants resulted in higher expression of several heat-responsive genes such as HSFA2, HSP101, HSP70, HSP70B, GA3OX1, HSP90.1, and ATERDJ3A as well as heat memory genes; APX2 (ascorbate peroxidase 2), HSP18.2, H3K4me3 (epigenetic modification to the DNA packaging protein Histone H3) (Lämke et al., 2016; Sedaghatmehr et al., 2016). In a study, increased expression of heat shock protein gene HSP17.8 and Dehydration-responsive gene DREB1 and decreased expression of POD47 and FeSOD have been reported for fungal (*Aspergillus aculeatus*) conferred heat stress tolerance in *Lolium perenne* grass (Li X. et al., 2021). Khan et al. (2020b) reported that GmLAX3 and GmAKT2, the key genes involved in the regulation of the ABA-dependent pathway, were downregulated initially but upregulated after 5 and 10 days in the inoculated soybean plant under thermal stress. The expression of these genes may be linked with plant stress response by modulating auxin and ABA signaling, decreasing ROS production and enhancing K^+^ gradients, which are critical in plant tissues under various stresses (Zhou et al., 2014; Chai et al., 2016). The *B. velezensis* inoculation induced an increased abiotic stress tolerance, and the pattern of metabolic modulation and abundance of several proteins were found to be similar in wheat challenged with different abiotic stress factors such as heat, cold, and drought (Abd El-Daim et al., 2019).

**FIGURE 7:**
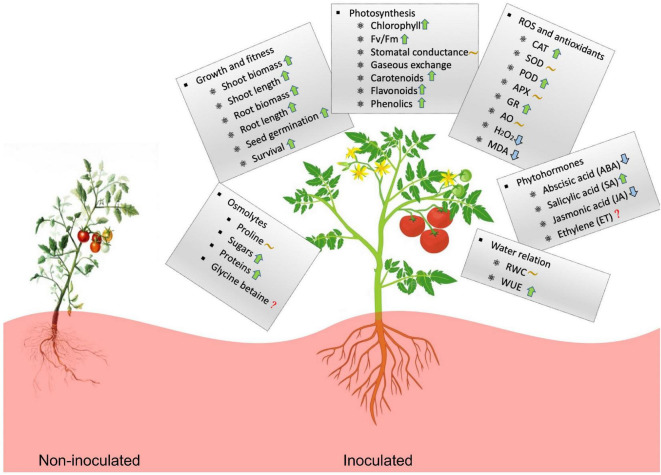
Illustration of the effects of microbial inoculation on plant physiological activity and stress tolerance under high temperature. The upward arrow (↑) indicates increase of the value for microbial-treated plants compared to non-treated plants whereas, downward arrow (↓) indicates decease, wavy line (∼) indicates the effect is variable and the question mark (?) unavailable information.

Taken together, high-temperature influences plant growth by affecting photosynthesis, osmotic balance, enzymatic activities, and metabolic activities. Microbial symbionts consistently help plants reduce the impact of thermal stress by manipulating physiological processes (Figure 7). Microbe-induced plant heat stress tolerance has broad ecological and agricultural implications. In some regions, increased stress tolerance can result in higher crop yield under higher temperature environments. Continued interest in the microbiome and their functional roles in uncovering the underlying mechanisms of plant-microbe interaction appears well-justified.

## Future Direction and Conclusion

Despite the evidence of global warming and the importance of heat stress on plant ecology and productivity (Wahid et al., 2007; Teixeira et al., 2013; Jagadish et al., 2021) and the increasing evidence of microbial functions of plant stress tolerance (Table 1 and Figure 1), the current review here identified a significant lack in our knowledge on microbial conferred plant performance and productivity under high-temperature conditions. Apparently, the most crucial of these is the lack of experiments that demonstrate how do microbes actually influence plant responses in the natural environment. Most of the investigations reviewed here were carried out under controlled environmental conditions and on cultivated host species. Therefore, experiments focusing on the impacts of symbionts on wild and crop plants exposed to heat stresses in the natural environment are required. In a recent paper, Ballesteros et al. (2020) analyzed metatranscriptomic data from the Antarctic plant *Colobanthus quitensis*, highlighting that the plant-associated fungal community provides beneficial effects, such as increased plant growth, tolerance to abiotic stress, and higher leaf carbon gain, when plants are grown under a simulated global warming scenario. The reports from these investigations should include mean values of response variables, measures of variance, and replication and preferably provide the actual dataset as the Supplementary Data so that these data can be used in future meta-analyses. Although it was shown that increasing temperatures due to climate change might negatively impact the outcomes of symbioses (Kiers et al., 2010), the current meta-analyses indicated that microbial symbionts have more positive effects on plant performance under heat stress than under normal conditions. It is not clear why microbial inoculation exerts its effects in more stressed conditions than under normal temperatures conditions, but putatively, this is associated with plant stress acclimation at the molecular or cellular levels. Tolerance of non-colonized plants to heat stress is well studied, and the reports suggested that the mechanisms are associated with enhanced expression of a variety of heat shock proteins, other related proteins, production of ROS, and induction of mitogen-activated protein kinase (MAPK) and calcium-dependent protein kinase (CDPK) cascades, and, most importantly, chaperone signaling and transcriptional activation (Wahid et al., 2007). As previously shown for abiotic stresses such as freezing and drought and assumed for microbial conferred cold adaptation (Knight and Knight, 2001; Acuna-Rodriguez et al., 2020), some kind of cross-talk between the proteins common to signaling pathways are present for responses to heat and microbial colonization leading to enhanced plant metabolism. The involvement of microbial elicitors at the plant cell surface resulting in increases in cytosolic Ca2^+^ concentrations could be another possibility (Sanders et al., 1999; Acuna-Rodriguez et al., 2020). However, more research is needed to establish whether these or similar mechanisms might explain the beneficial effects of microbial symbionts on plants at higher temperatures. As discussed by Acuna-Rodriguez et al. (2020), less attention has been paid toward understanding the differences in the optimum growth temperatures of microbial symbionts and their plant hosts. For instance, in the *Epichloe* symbiosis, the seemingly decreased plant performance at low temperatures in the natural conditions could be that the minimum temperatures requirement of the endophytes being higher than those of their hosts (Casler and Van Santen, 2008; Wäli et al., 2008). Extensive surveys to identify novel microbial symbionts capable of improving plant performance under stress are advocated. Isolation of naturally occurring plant and soil microbiomes and screening them for potential symbiotic benefits is a continuous process. Developing a rapid but robust screening method is important so that a large collection of microbes can be tested for their effects, and the results should be supported by field experiments in order to translate the research into the field. A few surveys focused on endophytes collected from hot environments, and some of the endophytes tested were found to grow at high temperatures (Zhou et al., 2015; Dastogeer et al., 2019). Studies on other components of the plant microbiome such as algae, protists, and viruses revealed that they are also present in the tissues or in the rhizosphere of many plant species and provide some fitness benefits (Roossinck, 2015; Gao et al., 2019; Dastogeer et al., 2020a; Dumack and Bonkowski, 2021; Lee and Ryu, 2021). For example, rhizosphere protists altered the metabolites of the maize plant, particularly, polyols. This alteration is presumably to be associated with reduction of the impact of stress caused due to grazing (Kuppardt et al., 2018). A plant virus, yellowtail flower mild mottle virus (YTMMV, genus Tobamovirus), enhanced drought stress tolerance in *Nicotiana benthamiana* plants by modulating its metabolic compounds (Dastogeer et al., 2018). However, the effect of many of the microbiome components has received less attention. We also suggest studies that consider microbial symbionts on plant heat tolerance should take into account the involvement of secondary metabolites because many microbes utilize these compounds in the interaction with plants and with environments (Zaynab et al., 2018; Ullah et al., 2020). Another important area of research, but has so far been neglected, is to study the multi-partite interaction of microbes and their impact on stress tolerance, such as thermal tolerance. It was found that a fungus can increase the heat tolerance of a plant only in the presence of a virus within it, whereas neither virus nor the plant separately could confer this benefit. Therefore, a three-way symbiosis is required for thermal tolerance. In nature, plants do not remain in isolation but in continuous interaction with a wide variety of microbes (Marquez et al., 2007). In addition to microbe-plant interaction, microbe-microbe and microbe-environment interaction should be taken into account to understand the stable functions of a particular microbe in its potential application in agricultural settings, which is a dynamic environment and subject to continuous changes (Dastogeer et al., 2020a).

## Author Contributions

KD developed the concept, performed the database search, partially collected the data, analyzed and interpreted the results, and wrote the draft. MZ, MR, and MS extracted the data, produced the graphs, discussed the results, and revised the manuscript. AC produced the table and discussed the results. All authors approved the final version of the manuscript.

## Conflict of Interest

The authors declare that the research was conducted in the absence of any commercial or financial relationships that could be construed as a potential conflict of interest.

## Publisher’s Note

All claims expressed in this article are solely those of the authors and do not necessarily represent those of their affiliated organizations, or those of the publisher, the editors and the reviewers. Any product that may be evaluated in this article, or claim that may be made by its manufacturer, is not guaranteed or endorsed by the publisher.
