# Supplementary material for: Microbe-Mediated Thermotolerance in Plants and Pertinent Mechanisms- A Meta-Analysis and Review
Source: Front Microbiol. 2022 Mar 7;13:833566. doi: 10.3389/fmicb.2022.833566 (PMC8940538; doi:10.3389/fmicb.2022.833566)
Supplement: Supplementary file 1 [file Table_1.DOCX]

Supplementary table 1 (ST1): Peer-reviewed original research articles used in the meta-analysis to identify the overall effects and mechanisms of microbe-mediated plant heat stress tolerance

(Eerens et al., 1998; Martin and Stutz, 2004; Ali et al., 2009; Bunn et al., 2009; Morsy et al., 2010; Ali et al., 2011; Zhu et al., 2011; Hubbard et al., 2012; Khan et al., 2012a; Khan et al., 2012b; Spiers et al., 2012; Khan et al., 2013; Maya and Matsubara, 2013; Abd El-Daim et al., 2014; Hubbard et al., 2014; Khan et al., 2015; Tian et al., 2015; Waqas et al., 2015; Cabral et al., 2016; Hennessy et al., 2016; Abd El-Daim et al., 2018; Ali et al., 2018; Duc et al., 2018; Ismail et al., 2018; Issa et al., 2018; Mathur et al., 2018; Sangamesh et al., 2018; Sarkar et al., 2018; Abd El-Daim et al., 2019; Ali et al., 2019; Ismail et al., 2019; Yeasmin et al., 2019; Bruno et al., 2020; Ismail et al., 2020a; Ismail et al., 2020b; Ismail et al., 2020c; Khan et al., 2020a; Khan et al., 2020b; Mathur and Jajoo, 2020; Mukhtar et al., 2020; Shekhawat et al., 2020; Li et al., 2021)

Abd El-Daim, I.A., Bejai, S., Fridborg, I., and Meijer, J. (2018). Identifying potential molecular factors involved in Bacillus amyloliquefaciens 5113 mediated abiotic stress tolerance in wheat. *Plant Biology* 20(2)**,** 271-279. doi: 10.1111/plb.12680.

Abd El-Daim, I.A., Bejai, S., and Meijer, J. (2014). Improved heat stress tolerance of wheat seedlings by bacterial seed treatment. *Plant and Soil* 379(1-2)**,** 337-350. doi: 10.1007/s11104-014-2063-3.

Abd El-Daim, I.A., Bejai, S., and Meijer, J. (2019). Bacillus velezensis 5113 induced metabolic and molecular reprogramming during abiotic stress tolerance in wheat. *Scientific reports* 9(1)**,** 1-18.

Ali, A.H., Abdelrahman, M., Radwan, U., El-Zayat, S., and El-Sayed, M.A. (2018). Effect of Thermomyces fungal endophyte isolated from extreme hot desert-adapted plant on heat stress tolerance of cucumber. *Applied Soil Ecology* 124**,** 155-162. doi: 10.1016/j.apsoil.2017.11.004.

Ali, A.H., Radwan, U., El-Zayat, S., and El-Sayed, M.A. (2019). The role of the endophytic fungus, Thermomyces lanuginosus, on mitigation of heat stress to its host desert plant Cullen plicata. *Biologia Futura* 70(1)**,** 1-7. doi: 10.1556/019.70.2019.01.

Ali, S.Z., Sandhya, V., Grover, M., Kishore, N., Rao, L.V., and Venkateswarlu, B. (2009). Pseudomonas sp. strain AKM-P6 enhances tolerance of sorghum seedlings to elevated temperatures. *Biology and Fertility of Soils* 46(1)**,** 45-55.

Ali, S.Z., Sandhya, V., Grover, M., Linga, V.R., and Bandi, V. (2011). Effect of inoculation with a thermotolerant plant growth promoting Pseudomonas putida strain AKMP7 on growth of wheat (Triticum spp.) under heat stress. *Journal of Plant Interactions* 6(4)**,** 239-246.

Bruno, L.B., Karthik, C., Ma, Y., Kadirvelu, K., Freitas, H., and Rajkumar, M. (2020). Amelioration of chromium and heat stresses in Sorghum bicolor by Cr6+ reducing-thermotolerant plant growth promoting bacteria. *Chemosphere* 244**,** 125521.

Bunn, R., Lekberg, Y., and Zabinski, C. (2009). Arbuscular mycorrhizal fungi ameliorate temperature stress in thermophilic plants. *Ecology* 90(5)**,** 1378-1388. doi: Doi 10.1890/07-2080.1.

Cabral, C., Ravnskov, S., Tringovska, I., and Wollenweber, B. (2016). Arbuscular mycorrhizal fungi modify nutrient allocation and composition in wheat (Triticum aestivum L.) subjected to heat-stress. *Plant and Soil* 408(1-2)**,** 385-399. doi: 10.1007/s11104-016-2942-x.

Duc, N.H., Csintalan, Z., and Posta, K. (2018). Arbuscular mycorrhizal fungi mitigate negative effects of combined drought and heat stress on tomato plants. *Plant Physiology and Biochemistry* 132**,** 297-307.

Eerens, J.P.J., Lucas, R.J., Easton, S., and White, J.G.H. (1998). Influence of the endophyte (Neotyphodium lolii) on morphology, physiology, and alkaloid synthesis of perennial ryegrass during high temperature and water stress. *New Zealand Journal of Agricultural Research* 41(2)**,** 219-226. doi: Doi 10.1080/00288233.1998.9513305.

Hennessy, L.M., Popay, A.J., Finch, S.C., Clearwater, M.J., and Cave, V.M. (2016). Temperature and plant genotype alter alkaloid concentrations in ryegrass infected with an Epichloë endophyte and this affects an insect herbivore. *Frontiers in plant science* 7**,** 1097.

Hubbard, M., Germida, J., and Vujanovic, V. (2012). Fungal endophytes improve wheat seed germination under heat and drought stress. *Botany-Botanique* 90(2)**,** 137-149. doi: 10.1139/B11-091.

Hubbard, M., Germida, J.J., and Vujanovic, V. (2014). Fungal endophytes enhance wheat heat and drought tolerance in terms of grain yield and second-generation seed viability. *Journal of Applied Microbiology* 116(1)**,** 109-122. doi: 10.1111/jam.12311.

Ismail, Hamayun, M., Hussain, A., Iqbal, A., Khan, S.A., and Lee, I.-J. (2020a). Aspergillus niger boosted heat stress tolerance in sunflower and soybean via regulating their metabolic and antioxidant system. *Journal of Plant Interactions* 15(1)**,** 223-232.

Ismail, Hamayun, M., Hussain, A., Iqbal, A., Khan, S.A., and Lee, I.J. (2018). Endophytic Fungus Aspergillus japonicus Mediates Host Plant Growth under Normal and Heat Stress Conditions. *Biomed Research International* 2018. doi: 10.1155/2018/7696831.

Ismail, Hamayun, M., Hussain, A., Khan, S.A., Iqbal, A., and Lee, I.J. (2020b). An endophytic fungus Aspergillus violaceofuscus can be used as heat stress adaptive tool for Glycine max L. and Helianthus annuus L. *Journal of Applied Botany and Food Quality* 93**,** 112-+. doi: 10.5073/Jabfq.2020.093.014.

Ismail, Hussain, A., Mehmood, A., Qadir, M., Husna, H., Iqbal, A., et al. (2020c). Thermal Stress Alleviating Potential of Endophytic Fungus Rhizopus Oryzae Inoculated to Sunflower (Helianthus Annuus L.) and Soybean (Glycine Max L.). *Pakistan Journal of Botany* 52(5)**,** 1857-1865. doi: 10.30848/Pjb2020-5(10).

Ismail, H., Muhammad, Hussain, A., Afzal Khan, S., Iqbal, A., and Lee, I.-J. (2019). Aspergillus flavus promoted the growth of soybean and sunflower seedlings at elevated temperature. *BioMed research international* 2019.

Issa, A., Esmaeel, Q., Sanchez, L., Courteaux, B., Guise, J.-F., Gibon, Y., et al. (2018). Impacts of Paraburkholderia phytofirmans strain PsJN on tomato (Lycopersicon esculentum L.) under high temperature. *Frontiers in plant science* 9**,** 1397.

Khan, A.L., Hamayun, M., Radhakrishnan, R., Waqas, M., Kang, S.-M., Kim, Y.-H., et al. (2012a). Mutualistic association of Paecilomyces formosus LHL10 offers thermotolerance to Cucumis sativus. *Antonie van Leeuwenhoek* 101(2)**,** 267-279.

Khan, A.L., Hamayun, M., Waqas, M., Kang, S.M., Kim, Y.H., Kim, D.H., et al. (2012b). Exophiala sp.LHL08 association gives heat stress tolerance by avoiding oxidative damage to cucumber plants. *Biology and Fertility of Soils* 48(5)**,** 519-529. doi: 10.1007/s00374-011-0649-y.

Khan, A.L., Kang, S.M., Dhakal, K.H., Hussain, J., Adnan, M., Kim, J.G., et al. (2013). Flavonoids and amino acid regulation in Capsicum annuum L. by endophytic fungi under different heat stress regimes. *Scientia Horticulturae* 155**,** 1-7. doi: 10.1016/j.scienta.2013.02.028.

Khan, A.L., Waqas, M., and Lee, I.-J. (2015). Resilience of Penicillium resedanum LK6 and exogenous gibberellin in improving Capsicum annuum growth under abiotic stresses. *Journal of plant research* 128(2)**,** 259-268.

Khan, M.A., Asaf, S., Khan, A.L., Jan, R., Kang, S.-M., Kim, K.-M., et al. (2020a). Extending thermotolerance to tomato seedlings by inoculation with SA1 isolate of Bacillus cereus and comparison with exogenous humic acid application. *PLoS One* 15(4)**,** e0232228.

Khan, M.A., Asaf, S., Khan, A.L., Jan, R., Kang, S.M., Kim, K.M., et al. (2020b). Thermotolerance effect of plant growth-promotingBacillus cereusSA1 on soybean during heat stress. *Bmc Microbiology* 20(1). doi: 10.1186/s12866-020-01822-7.

Li, X., Zhao, C., Zhang, T., Wang, G., Amombo, E., Xie, Y., et al. (2021). Exogenous Aspergillus aculeatus Enhances Drought and Heat Tolerance of Perennial Ryegrass. *Frontiers in microbiology* 12**,** 307.

Martin, C.A., and Stutz, J.C. (2004). Interactive effects of temperature and arbuscular mycorrhizal fungi on growth, P uptake and root respiration of Capsicum annuum L. *Mycorrhiza* 14(4)**,** 241-244. doi: 10.1007/s00572-003-0261-6.

Mathur, S., and Jajoo, A. (2020). Arbuscular mycorrhizal fungi protects maize plants from high temperature stress by regulating photosystem II heterogeneity. *Industrial Crops and Products* 143. doi: 10.1016/j.indcrop.2019.111934.

Mathur, S., Sharma, M.P., and Jajoo, A. (2018). Improved photosynthetic efficacy of maize (Zea mays) plants with arbuscular mycorrhizal fungi (AMF) under high temperature stress. *Journal of Photochemistry and Photobiology B: Biology* 180**,** 149-154.

Maya, M.A., and Matsubara, Y. (2013). Influence of arbuscular mycorrhiza on the growth and antioxidative activity in cyclamen under heat stress. *Mycorrhiza* 23(5)**,** 381-390. doi: 10.1007/s00572-013-0477-z.

Morsy, M.R., Oswald, J., He, J., Tang, Y., and Roossinck, M.J. (2010). Teasing apart a three-way symbiosis: transcriptome analyses of Curvularia protuberata in response to viral infection and heat stress. *Biochem Biophys Res Commun* 401(2)**,** 225-230. doi: 10.1016/j.bbrc.2010.09.034.

Mukhtar, T., Rehman, S.U., Smith, D., Sultan, T., Seleiman, M.F., Alsadon, A.A., et al. (2020). Mitigation of Heat Stress in Solanum lycopersicum L. by ACC-deaminase and Exopolysaccharide Producing Bacillus cereus: Effects on Biochemical Profiling. *Sustainability* 12(6). doi: 10.3390/su12062159.

Sangamesh, M., Jambagi, S., Vasanthakumari, M., Shetty, N.J., Kolte, H., Ravikanth, G., et al. (2018). Thermotolerance of fungal endophytes isolated from plants adapted to the Thar Desert, India. *Symbiosis* 75(2)**,** 135-147.

Sarkar, J., Chakraborty, B., and Chakraborty, U. (2018). Plant growth promoting rhizobacteria protect wheat plants against temperature stress through antioxidant signalling and reducing chloroplast and membrane injury. *Journal of Plant Growth Regulation* 37(4)**,** 1396-1412.

Shekhawat, K., Sheikh, A., Mariappan, K., Jalal, R., and Hirt, H. (2020). Enterobacter sp. SA187 mediates plant thermotolerance by chromatin modification of heat stress genes. *bioRxiv*.

Spiers, D.E., Wax, L.E., Eichen, P.A., Rottinghaus, G.E., Evans, T.J., Keisler, D.H., et al. (2012). Use of different levels of ground endophyte-infected tall fescue seed during heat stress to separate characteristics of fescue toxicosis. *Journal of Animal Science* 90(10)**,** 3457-3467. doi: 10.2527/jas.2012-5099.

Tian, Z.P., Huang, B.R., and Belanger, F.C. (2015). Effects of Epichloe festucae Fungal Endophyte Infection on Drought and Heat Stress Responses of Strong Creeping Red Fescue. *Journal of the American Society for Horticultural Science* 140(3)**,** 257-264. doi: Doi 10.21273/Jashs.140.3.257.

Waqas, M., Khan, A.L., Shahzad, R., Ullah, I., Khan, A.R., and Lee, I.J. (2015). Mutualistic fungal endophytes produce phytohormones and organic acids that promote japonica rice plant growth under prolonged heat stress. *Journal of Zhejiang University-Science B* 16(12)**,** 1011-1018. doi: 10.1631/jzus.B1500081.

Yeasmin, R., Bonser, S.P., Motoki, S., and Nishihara, E. (2019). Arbuscular Mycorrhiza Influences Growth and Nutrient Uptake of Asparagus (Asparagus officinalis L.) under Heat Stress. *Hortscience* 54(5)**,** 846-850. doi: 10.21273/Hortsci13587-18.

Zhu, X.C., Song, F.B., Liu, S.Q., and Liu, T.D. (2011). Effects of arbuscular mycorrhizal fungus on photosynthesis and water status of maize under high temperature stress. *Plant and Soil* 346(1-2)**,** 189-199. doi: 10.1007/s11104-011-0809-8.

Supplementary table 2 (ST2): Publication bias as tested by Eggers' test of significant of Funnel plot asymmetry. Values of SMD that are bolded indicates that the effect size was significant at least p <0.05.

| Biomass | Stress conditions | SMD | Eggers' test | SMD (after trim fill) |
| --- | --- | --- | --- | --- |
| Shoot dry biomass | Non-stressed | **0.4395** | 0.1136 |  |
|  | Stressed | **0.7677** | 0.3783 |  |
| Root dry biomass | Non-stressed | 0.6046 | 0.0191 |  |
|  | Stressed | **0.4728** | 0.0005 | 0.2805 |
| Shoot length | Non-stressed | **1.9070** | 0.0009 | **1.3906** |
|  | Stressed | **1.9891** | <0.0001 | **1.2206** |
| Root length | Non-stressed | **0.6765** | 0.0033 | 0.4106 |
|  | Stressed | 0.6739 | 0.0042 | -0.1585 |
| RWC | Non-stressed | 0.9610 | 0.3076 |  |
|  | Stressed | 1.6640 | 0.3513 |  |
| Photosynthesis | Non-stressed | **1.7944** | 0.0301 |  |
|  | Stressed | **2.8008** | 0.0148 |  |
| Total Chlorophyll | Non-stressed | **0.9443** | 0.1111 |  |
|  | Stressed | **1.3665** | 0.0099 | **0.8653** |
| Stomatal conductance | Non-stressed | 1.1473 | 0.2921 |  |
|  | Stressed | 1.2158 | 0.2234 |  |
| Fv/Fm | Non-stressed | 0.1339 | 0.0635 |  |
|  | Stressed | **0.9496** | 0.0114 |  |
| CAROTENOIDs | Non-stressed | 0.5212 | 0.0219 |  |
|  | Stressed | **1.1909** | <0.0001 | **0.8057** |
| FLAVONOIDS | Non-stressed | 0.7419 | 0.0024 | 0.4927 |
|  | Stressed | **0**.**9584** | <0.0001 | 0.6206 |
| PHENOLICS | Non-stressed | **1.1858** | <0.0001 | **0.8336** |
|  | Stressed | **0.9356** | <0.0001 | 0.6077 |
| ABA | Non-stressed | -0.5127 | 0.0448 |  |
|  | Stressed | **-1.7982** | <0.0001 | **-1.5027** |
| SA | Non-stressed | 0.5258 | 0.7146 |  |
|  | Stressed | **2.7535** | 0.0003 | **2.7535** |
| Proline | Non-stressed | 0.0391 | 0.0892 |  |
|  | Stressed | 0.3630 | 0.0328 |  |
| SS | Non-stressed | **1.3339** | <0.0001 | 0.8301 |
|  | Stressed | **1.1701** | 0.2073 |  |
| AAO | Non-stressed | 0.0801 | 0.4825 |  |
|  | Stressed | -0.9161 | 0.0372 |  |
| APX | Non-stressed | 0.6557 | 0.1149 |  |
|  | Stressed | 0.5828 | 0.0745 |  |
| CAT | Non-stressed | 0.8355 | 0.0235 |  |
|  | Stressed | **1.3388** | 0.1054 |  |
| GR | Non-stressed | **0.7780** | <0.0001 | **0.7780** |
|  | Stressed | **1.3347** | 0.0025 | **1.0089** |
| SOD | Non-stressed | 1.1597 | 0.2721 |  |
|  | Stressed | 1.7404 | .2169789 |  |
| POD | Non-stressed | 1.5799 | 0.135056 |  |
|  | Stressed | **3.3440** | 0.1301 |  |
| H2O2 | Non-stressed | **-0.7842** | <0.0001 | -0.4111 |
|  | Stressed | **-3.0699** | 0.0042 | **-2.5752** |
| MDA | Non-stressed | -0.1421 | 0.8211 |  |
|  | Stressed | -1.2738 | 0.0694 |  |
